# How do persons with dementia participate in decision making related to health and daily care? A multi-case study

**DOI:** 10.1186/1472-6963-12-241

**Published:** 2012-08-07

**Authors:** Kari Lislerud Smebye, Marit Kirkevold, Knut Engedal

**Affiliations:** 1 Faculty of Health and Social Work Studies, Ostfold University College, 1757, Halden, Norway; 2 Institute for Health and Society, Faculty of Medicine, University of Oslo, P.B. 1130, Blindern, 0318, Oslo, Norway; 3 Institute of Public Health, Aarhus University, Aarhus, Denmark; 4 Norwegian Centre for Aging and Health, Oslo University Hospital HF, Ulleval, Bygning 37, 407, Oslo, Norway

## Abstract

**Background:**

Many countries have passed laws giving patients the right to participate in decisions about health care. People with dementia cannot be assumed to be incapable of making decisions on their diagnosis alone as they may have retained cognitive abilities.

The purpose of this study was to gain a better understanding of how persons with dementia participated in making decisions about health care and how their family carers and professional caregivers influenced decision making.

**Methods:**

This Norwegian study had a qualitative multi-case design. The triad in each of the ten cases consisted of the person with dementia, the family carer and the professional caregiver, in all 30 participants. Inclusion criteria for the persons with dementia were: (1) 67 years or older (2) diagnosed with dementia (3) Clinical Dementia Rating score 2, moderate dementia; (3) able to communicate verbally. The family carers and professional caregivers were then asked to participate.

A semi-structured interview guide was used in interviews with family carers and professional caregivers. Field notes were written after participant observation of interactions between persons with dementia and professional caregivers during morning care or activities at a day centre. How the professional caregivers facilitated decision making was the focus of the observations that varied in length from 30 to 90 minutes. The data were analyzed using framework analysis combined with a hermeneutical interpretive approach.

**Results:**

Professional caregivers based their assessment of mental competence on experience and not on standardized tests. Persons with dementia demonstrated variability in how they participated in decision making. Pseudo-autonomous decision making and delegating decision making were new categories that emerged. Autonomous decision making did occur but shared decision making was the most typical pattern. Reduced mental capacity, lack of available choices or not being given the opportunity to participate led to non-involvement. Not all decisions were based on logic; personal values and relationships were also considered.

**Conclusions:**

Persons with moderate dementia demonstrated variability in how they participated in decision making. Optimal involvement was facilitated by positioning them as capable of influencing decisions, assessing decision-specific competence, clarifying values and understanding the significance of relationships and context.

## Background

The Western ideal of autonomy in health matters has led to many countries passing laws giving patients the right to participate in deciding about their treatment and care. Dementia is a disease that progresses over many years, resulting in gradual decline in decision making ability. However, persons with dementia cannot be assumed to be incapable of making decisions on the basis of their diagnosis alone as they may have retained abilities [1,2]. In this study, the focus was on how the right to participate in decision making was exercised in dementia care. This constitutes an enormous challenge as dementia will affect an increasing number of people in the coming years. It is estimated that currently 24.3 million people worldwide have dementia [3].

Optimizing the potential for decision making in persons with dementia contributes to the maintenance of identity, well-being and quality of life [4-6] in addition to promoting dignity, integrity and personhood [7-9]. Using retained cognitive abilities prevents excess disability [10-12]. Excluding persons from taking part in decisions can result in depression, frustration and anger, exacerbating the effects of the neuropathology already in existence, rendering the person even more debilitated [1].

There is evidence that people with dementia in institutions as well as people living in their own homes in the community are excluded from making decisions about their lives [13-15]. Some do not wish to participate in decision making [16], whilst others have the will and the ability to participate but are not given the chance [1,17].

### Competence and levels of involvement in decision making

The progress of dementia symptoms such as memory loss, communication problems and slowed processing speed, reduced mental competence in persons with dementia [17-19]. The McArthur Competence Assessment Tool for Treatment (MacCat-T) [20] is the most widely acclaimed test developed for assessing cognitive competence [21]. This test is based on the functional abilities of understanding relevant information, appreciating the significance of the information for one’s own situation, reasoning by considering alternatives and finally expressing choice. These cognitive abilities are prerequisites for making autonomous decisions and an assessment results in concluding if a person is competent or not.

Rather than assessing a person’s general competence, it can be more feasible to determine decision-specific competence [22]. Decisions vary in complexity according to the nature and consequences of the decision. Decisions about daily activities are less complex than deciding about financial matters. Greater competency is required for more complex decisions and thus influences how autonomous decision making can be [23]. Continuous assessment is another consideration as a person with dementia may have intermittent competency as fluctuating episodes of lucidity do occur [24].

However, not all decisions are based on logic and deliberation; many are intuitive and based on emotions, needs, values, preferences or habits [25,26]. Even with significant cognitive decline, persons with dementia can still be “valuers” i.e. they can, on the basis of what they unreflectively identify with, still evaluate, interpret and derive meaning in their lives [1,27]. The ability to value is independent of cognition and the pertinent question is if the person can still value and experience. Persons with mild to moderate dementia are able to state their preferences and be involved in decision making [28,29] and to a certain degree they are able to state their preferences consistently [30-33].

McCormack [34] found that it was more important for older patients to be informed and their values and preferences considered than that they made the actual decisions. Knowing the person with dementia enables others to understand their values and individual decision making patterns [35,36].

Yet, research is inconsistent on how well family carers’ preferences coincide with those of their family member with dementia [16,37-39]. This makes it all the more important to ascertain individual values, interests and preferences of the person with dementia.

Autonomous decision making as an individual right is increasingly being questioned [40,41] as people are dependent on each other and decisions are negotiated to ensure the interests of those involved [42-44]. Decisions are made in the context of relationships and what meaning situations hold for the persons involved [22].

This study draws on the work of Thompson’s model for participating in decision making [45]. Based on individual and group interviews with adult and mentally competent patients, Thompson studied patient involvement in situations such as consultations, treatments and continuing care. He identified five levels of involvement: 0) non-involvement where patients are passive recipients of care and treatment (1) information seeking/information receptive, considered an elementary stage of involvement and a requirement for being able to take part in decision making (2) information dialogue incorporating exchange of information between patient and clinicians (3) shared decision making where patients and clinicians cooperate in finding solutions and patients experience that their opinions are considered for decision making (4) autonomous decision making when the patient makes independent decisions. The patient’s ability to be involved varied from low to high as described at the different levels. Even though persons with dementia were not involved in Thompson’s study, his taxonomy was considered appropriate as it was assumed that they participated at different levels. Different participation levels are followed by gradation of power in decision making [46]. Since people with dementia are dependent on others, this affects how power is exerted in relationships and in decision making.

The present body of knowledge is fragmented and incomplete. Few studies have specifically explored how older people with dementia participate in decision making [22,42]. A main reason is that autonomy, participation and decision making are theoretical and multifaceted concepts which are difficult to operationalize and measure in practice. Thus, empirically-based research is needed to add to existing knowledge on how persons with dementia participate in decision making processes in dementia care [22,23,47]. Family carers and professional caregivers also influence the decision making of persons with dementia and combining these triadic perspectives is recommended [48].

This study was based on actual decisions as the use of hypothetical vignettes often used for testing decision making abilities was not found meaningful. In dementia research care decisions need to be studied in a real-life context in order to understand the processes involved and the interdependence between people with dementia, family carers and professional caregivers [34,43].

The purpose of the study was to understand how persons with dementia participated in decision making related to health and daily care and how their family carers and professional caregivers influenced decision making.

## Methods

This Norwegian study had a qualitative multi-case design and was conducted in three municipalities. The triad in each of the ten cases consisted of the person with dementia, family carer and professional caregiver (primary care nurse); in all 30 participants. Inclusion criteria for the persons with dementia were: (1) 67 years or older (2) diagnosed with dementia (3) Clinical Dementia Rating [49] score 2, i.e. moderate dementia; (3) able to communicate verbally. Diversity was promoted through purposive sampling. Three persons lived independently, two persons lived with close relatives and five persons had moved to sheltered housing or to a nursing home. The mean age was 83 years and only two were men. The group of family carers consisted of three spouses, two siblings, three adult children, a daughter-in-law and a niece. Only four family caregivers were men. The professional caregivers consisted of two registered nurses, six enrolled nurses and two nurse aids, all women.

Twenty-six older persons were asked to participate in the study and the main reasons for not being included were: no diagnosis, did not wish to participate or their family thought it would be too stressful for them.

Three types of decisions with varying complexity and consequences were studied: decisions concerning daily activities, medical care and moving to sheltered housing or a nursing home.

### Data collection

A semi-structured interview guide was used in interviews with family carers and professional caregivers. Open-ended questions were asked such as how they assessed decision-making competence, what facilitated or hindered the person’s decision making and their experiences of collaboration and coordination of services. The interviews lasted approximately one hour and were audio-recorded and transcribed verbatim. Field notes were written after participant observation of interactions between persons with dementia and professional caregivers during morning care or activities at a day centre. How the professional caregivers facilitated decision making was the focus of the observations that varied in length from 30 to 90 minutes. Data from the interviews and observations were collected by one person (KLS). Supplementary data sources were nursing and medical records and tests (Mini-Mental-Status-Examination [50], Neuropsychiatric Inventory [51] and Disability Assessment for Dementia [52]. Because of the dementia trajectory, all data in each case were collected in the course of 1–2 days. Data were collected from October 2007 to January 2009 (Table 1). 

**Table 1:** Sample: persons with dementia, family carers and professional caregivers

**Case**	**MMSE**	**DAD**	**NPI – severity of symptoms**	**Residence**	**Services**	**Family carer**	**Prof. care-giver**
**Mr**	23	74%	Agitation: 3	Nursing home	Special dementia unit	Spouse	EN
**A**			Anxiety: 3				
			Apathy: 3				
			Abnormal motor behaviour: 2				
**Mrs**	20	73%	Depression: 3	Flat - lived alone	Home nursing 3 times/day; day centre for persons with dementia 5 days/week	Sister	RN
**B**							
**Mrs**	22	80%	Depression: 4	Sheltered housing	Home nursing 2 times/day; general day centre 4 days/week	Daughter-in-law	EN
**C**							
**Mrs**	22	85%	Agitation: 4	Sheltered housing	Home nursing 3 times/day; general day centre 4 days/week; housecleaning and laundry 1.5 hour/fortnight	Son	EN
**D**			Depression: 3				
			Apathy: 3				
			Eating behaviour: 3				
**Mrs**	21	81%	Depression: 4	Nursing home	General ward	Son	NA
**E**			Anxiety: 4				
			Eating behaviour: 4				
**Mr**	21	69%	Loss of conditioned reflex: 2	Flat - lived with wife	General day centre 2 days/week	Spouse	EN
**F**							
**Miss**	21	78%.	Depression: 3	House - lived alone	Home nursing 3 times/day; general day centre 2 days/week; housecleaning 1 hour/week; meals-on-wheels 3 times/week; weekly visitor	Brother	EN
**G**			Anxiety: 2				
**Miss**	21	68%	0	Sheltered housing	Special dementia unit	Niece	NA
**H**							
**Mrs**	20	71%	Delusions: 2	House - lived alone	Home nursing 2–3 times/day; day centre for persons with dementia 5 days/week; housecleaning 1,5 hours/fortnight	Daughter	EN
**I**			Eating behaviour: 3				
**Mrs**	20	80%	0	House - lived with husband	Day centre for persons with dementia 2 days/week	Spouse	RN
**J**							

Abbreviations.

MMSE – Minimal Mental State Examination – moderately demented 20–25 points, max. 30 points.

DAD – Disability Assessment for Dementia – measures initiative, capacity to plan and executive functioning (hygiene, dressing, continence, meals, using telephone, managing correspondence and financial matters, household chores and leisure activities).

NPI – Neuropsychiatric Inventory rates severity of psychiatric symptoms and behavioural disturbances.

Professional caregiver:

RN – registered nurse.

EN – enrolled nurse.

NA – nurse aid.

### Analysis

Data from interviews and observations were analyzed using framework analysis yet allowing new categories to emerge. This was combined with a hermeneutic interpretive approach [53,54]. Thompson’s taxonomy of participation levels [45] was a frame of reference. Framework analysis allowed for a priori as well as emergent codes to be used [55,56]. New emergent themes were used to update the framework when applied to understanding how persons with dementia participated in decision making.

The text from the interviews and observations was read thoroughly to identify preliminary main themes. The deductive approach with the use of Thompson’s framework [45], gave partial insight but the material revealed more complex ways of involvement. The analysis was supplemented by adding an inductive approach. Codes were grouped under themes with sub-headings. In each case it was registered how family carers and professional caregivers had influenced understanding, appreciation, reasoning and expression. Each case was analyzed before looking for common themes in all ten cases.

The analysis continued by reviewing the text, from parts to the whole, and vice versa, until themes were clarified [54,57]. With this reflective, spiral process, the categories of participation in decision making were derived. The data were read and analyzed by the first and second author until consensus concerning essential themes was reached.

### Ethical considerations

Staff working in nursing services, sheltered housing and nursing homes in the three municipalities were informed about the study and asked to identify patients meeting inclusion criteria. Patients were asked to participate after receiving information written in everyday language in a letter they could keep in their purse or pocket and could be reread to compensate for deficits in short-term memory. They were assured that non-consent would have no impact on services provided. It was a concern that they felt obliged to consent when asked by a caregiver on whom they were dependent. On the other hand, being asked by a known and trusted person reduced anxiety [58-60]. Family carers who helped the person with dementia on a regular basis were asked to participate and for consent concerning participation of the person with dementia. Consent was also sought on an ongoing basis (process consent) as the person with dementia was asked again on the observation day for participation consent. Beforehand, it was agreed on that if verbal or non-verbal expressions of discomfort were registered, the person would not be included in the study. However, this did not occur.

The research project was approved by the Regional Ethical Committee for Medical Research (reference number S-07181a) and the Norwegian Social Science Data Services (project number 17352).

### Trustworthiness

An attempt has been made to leave “a decision trail” so that verification strategies such as investigator responsiveness, methodological coherence, transparency and an analytic stance could be identified [61,62]. A framework analysis can be viewed as prescriptive, providing a systematic and visible structure to the analysis process, helping to maintain an audit trail and allowing flexibility for new themes to emerge. Triangulation of data added to the rigour of the study [56,63]. Different methods and multiple data sources contributed to more comprehensive descriptions of the complex process of decision making. Through participant observation non-verbal signs and details in the environment were registered, thus supplementing interview data.

Participation observation could influence behaviour but concealed observation was ethically no option. Pre-understandings were scrutinized as they influence how text develops during the interview and analysis [64,65]. Throughout the writing process, both authors discussed this issue repeatedly. At times the first author’s nursing pre-understanding led to focusing on therapeutic intervention and not primarily as a researcher attempting to interpret the text.

## Results

There was scarce evidence in patient’s records of mental competence being systematically tested even though all had taken a Mini-Mental-Status-Examination Results ranged from 20 to23. According to family carers, all but one person with dementia had been informed about their diagnosis; five persons understood what this entailed, two persons had partial insight and three persons had no insight.

In all ten cases, no extensive registration of life history was registered in the patient records. This limited the professional caregivers’ understanding of the person’s values and what different relationships meant to their patients.

There was considerable variability in how persons with dementia were involved in their own decisions. Pseudo-autonomous decision making and delegated decision were new categories that emerged. Shared decision making and autonomous decision making were identified. Persons with dementia were more autonomous in decisions about daily activities than in medical treatment or in deciding to move to sheltered housing or a nursing home. Shared decision making seemed to be the most typical pattern of decision making.

Non-involvement occurred when persons because of the trajectory of dementia were no longer capable of being involved, there were no choices available or they were not given the opportunity to participate. To illustrate different categories of involvement, examples have been selected according to three criteria: being illustrative of a particular category, offering a range of views where there was heterogeneity and being focused and succinct.

### Autonomous decision making

There were few autonomous decisions made by the participants. These were decisions concerning daily activities important for well-being but without serious consequences whatever decision was made. Family carers and professional caregivers ensured that persons were informed and checked to make sure that they understood what the information meant in their situation. They helped clarify the range of choices by simplifying their environments in such ways as keeping things tidy, removing irrelevant objects and keeping order by labelling drawers. Stating possible alternatives in a clear and concrete manner was at times necessary. Failing memory was compensated with aids/props to make options clearer. Narrowing the range of available choices because of limited powers of concentration and deliberation reduced confusion and promoted autonomous decision making.

The following illustrates an assessment of competence and autonomous decision making in choosing activities at a day centre. The professional caregiver (PC) was asked if Mrs J was capable of deciding in what activities she wished to partake.

"PC: “If explained what, why and how - she can join in deciding how she wants to participate - that’s my opinion. It is evident that she has a huge memory loss so she will not remember this afterwards but there and then she can assess the situation quite adequately.”"

"On one occasion the PC held up the local newspaper and announced that those wanting to join her for a reading session could follow her to the sitting-room. Another staff member stood by a table where baking equipment was laid out and explained that baking cookies was another option. Mrs J said she had already read the paper that morning and wanted to bake because she liked to and was familiar with baking."

Being perceived as a person capable of making decisions influenced Mrs J’s opportunities to participate. The staff at the day centre facilitated decision making by preparing activities and communicated with her verbally and non-verbally to make options clearer. They gave her time to ponder the alternatives. Family members had provided background information on each person, making it easier for staff to plan activities. Whilst reflecting on what activity to choose, Mrs J expressed her values and gave rational reasons for her decision.

According to her husband, Mrs J demonstrated a different pattern of decision making at home. Their relationship was strained and her husband said that when he made suggestions, she would often make an autonomous decision exactly the opposite of what he considered to be a wise decision. He said this often happened when they were together with family and friends and her behaviour embarrassed him. She felt that he ridiculed her in public when he so often disagreed with her. Her independent decisions seemed therefore to be a protest against her husband and a demonstration of autonomy, based on emotions such as anger and frustration and not so much rational reasoning. In her home Mrs J was negatively positioned by her husband as incapable of making decisions in contrast to how the professional caregivers at the day centre facilitated her participation.

### Pseudo-autonomous decision making

What appeared to be autonomous decision making could in fact be pseudo-autonomous if the prerequisites for the decision-making process were absent. Decisions were implicit rather than explicit. The presence or absence of dialogue was one of the discerning factors between autonomous and pseudo-autonomous decision making. The person with dementia had in some instances not been adequately informed and available situational choices not openly discussed. In some cases family carers and professional caregivers merely assumed they knew the person’s values and preferences without sufficient clarification and decisions were based on unclear or false premises. A consequence could be that the interests of others were prioritized instead of the interests of persons with dementia. This was illustrated in the following example:

"Mrs B was a quiet woman who trusted her family and did not oppose them. Moving to a nursing home had never been discussed openly with her and thus she had not made an explicit decision about her future. It was assumed that Mrs B wished to remain living next door to her sister. The sister had bought this flat for Mrs B so she could be close by to help her. Mrs B often expressed gratitude for her sister’s continuous and self-sacrificing help. The nursing services offered to help Mrs B move to a nursing home because, according to their assessment, she suffered from sensory deprivation in her present situation. The sister’s response to this offer was: “I’m not quite ready for that yet! … I always need something to do. If not, time passes by so slowly.”"

In this case Mrs B was not questioned directly about her views on moving to a nursing home. It was assumed she wanted to remain living in her own home. She often expressed her gratitude towards her sister for all her help in enabling her to continue living independently. However, it could be that Mrs B never expressed any wish of moving to a nursing home because it would imply that she did not appreciate or was critical of her sister’s efforts. This could be understood as wanting to comply with her sister’s need for someone to care for and enabling them to share each other’s company. The sister’s fear of boredom or loneliness could have been an underlying reason for helping Mrs B live in her home.

The primary care nurse later arranged for respite care in a nursing home and Mrs B thrived, evidenced by her being more awake, enjoying the company of other residents and gaining weight. In this case, pseudo-autonomous decision making entailed implicit rather than explicit decisions.

A contrasting case is Miss G who was very clear about what was important to her.

"PC: “She really wants to live in her own home. It means everything to her!”"

"Brother:“… Because of everything she did for my father, we will not pressure her. She can have it her way!”"

Knowing Miss G and her life history was necessary to understand her strong desire to live in the home her father had built and where she had cared for her ailing father for many years. She made an autonomous decision to live in her home but needed help from others to carry out her decision. Her brother was in a wheel-chair, yet he helped her as much as possible by paying his sister’s bills on the Internet, arranging for a neighbour to help with the snow and mow the lawn and called her on the days she was to attend the day centre. He supported her wish of living independently even though there were risks such as the possibility of fire or falling down stairs. Knowledge of the family history helped to understand how the norm of reciprocity influenced decisions that were made. The community nurses worked in partnership with the family and helpers came three to four times a day, helping her with her personal hygiene, housecleaning and shopping, they arranged for a voluntary visitor and organized transport to the day centre.

### Delegating decision making

This type of involvement was identified for major decisions and in matters concerning daily activities. Delegating responsibility to others emerged as a new category based on conscious decision making by persons with dementia concerning authorizing others to decide on their behalf. This was an active decision and not passive acceptance of letting others decide as seen in non-involvement. Some delegated authority to others on the spur of the moment; others did so after thorough deliberation but always to persons whom they trusted. Strong family bonds and social capital, accumulated by fulfilling family norms and obligations over the years, influenced to whom persons with dementia delegated authority. Some were delegated responsibility because of certain qualifications such as delegating responsibility for medical matters to a family member who was a nurse. She accompanied her relative to the doctor, bought medicine and monitored the effects and side-effects of medications.

A certain insight in their own limitations was a factor that triggered persons with dementia to delegate responsibility to others. They did not seem to completely trust their own judgement. During morning care in a nursing home, a resident was overheard to say to her primary caregiver: “*You decide. You think clearer than I do!”*

Miss H deliberately delegated responsibility to a family member. Her niece explained how she became her aunt’s main family carer:

"Niece: “Aunt H has sort of always been a part of our family… for every Christmas and for birthdays she has always come along so we are used to that…. There have been solid family bonds … so it has sort of been us through all these years…… When she retired she moved into the same housing complex where I live. That was probably a conscious decision she made when she retired…… Yes, by moving in there, I was made aware of this at a quite early stage. She has given me the responsibility and the role of family carer… I have expanded it as her needs … increased.”"

In this family social capital had been built through the years. As a seamstress Miss H had sewn clothes and helped her niece and nephews. She had been included in family gatherings and was considered part of the family. When she retired she chose to move close to her niece and before an operation she made arrangements for her niece to manage her finances. At first, authority was delegated to a specific domain but developed in time to global authority. The niece took on increased responsibility when necessary, based on her sense of duty and loyalty towards a family member who chose her as “guardian” when moving in next door.

### Shared decision making

Shared decision making was the most dominant pattern demonstrating that persons with dementia were dependent on others. They could have problems understanding information, they might not trust their own reasoning abilities or they needed more than the allotted time to consider options. Helpers compensated for lost abilities or facilitated the use of retained functions for example by offering support or reinforcing opinions when persons with dementia did not trust their own judgement, were ambiguous and had a hard time deciding what to do.

It was typical that there was an exchange of information and a questioning and answering pattern in the dialogue. Helpers who were aware of how important it was to ensure the flow of information, enquired about the person’s views, checked to make sure they understood the information and waited for answers. Questions were also used to remind persons of their options. In shared decision making, support was especially needed for reasoning.

Clarifying issues and having a shared understanding of the situation aided collaboration within the triad. Persons with dementia were consulted and their views and preferences influenced the decisions taken by family carers and professional caregivers. Decision making was a joint venture and negotiated within partnerships.

The following example shows shared decision making concerning a daily activity. In this situation Miss G and the professional caregiver had a common aim of making out a list for grocery shopping:

"PC: “If you … look into the refrigerator, then I will write….”"

"Miss G: “Butter, and then I need more cheese. There are eggs here, so I have plenty of that. Here is cream for coffee.”"

"PC: (looks to see if it is outdated) and says: “I think you should empty that out and then we can throw away the carton.”"

"Miss G: “But don’t you think it’s ok?”"

"PC: “No, I don’t think it’s wise to use it because it may be sour, which makes you sick in your stomach. Do you need any bread?”"

"Miss G: “Yes, I do. That bread [that she had for breakfast] was a little dry!”"

"PC: “You need something to drink. There is mineral water in the refrigerator. What about a half litre of milk?”"

"Miss G:”Yes, I need more milk.”"

The PC and Miss G both looked at what was in the refrigerator – a concrete and practical way for the person with dementia to see what was needed. The items on the shopping list were negotiated between the two. The PC explained that food would go bad if it was kept too long and that she could become ill if she ate it. Miss G had been thrifty all her life and questioned throwing away food. She had to consider alternatives and their consequences before deciding what to do. The PC gave cues all along and made the list as Miss G checked what was in the refrigerator and stated her preferences. This ensured that the shopping list was made out on her own terms even though she was dependent on help from the PC.

Another type of shared decision making was registered when caregivers referred to previous negotiations and agreements. In a specific situation, it was then not necessary to repeat these negotiations. To a certain extent this also gave the person with dementia control if they could recall the agreement or see written information. An example from morning care illustrates this:

"PC: It is Friday today and on Fridays we have an agreement about showering. Look at your plan for the week written on your board. Come let me help you!”"

"Mrs C could decide to accept or decline the offer."

Giving and sharing information was a key issue, also a challenging one. Professional caregivers compared informing persons with dementia as a *“balancing act”.* On the one hand they needed to explain matters thoroughly but on the other hand giving excessive information overwhelmed and confused the person. This was especially challenging when new concepts and ideas were introduced. It was difficult to comprehend that a credit card could be used instead of cash when shopping or that the use of plaster (Exelon) could improve memory.

A quote from a professional caregiver illuminates this:

"“It is a balancing act that never ends; explaining everything in a way that helps her to understand at the same time… you can not explain yourself to death either! Sometimes she says: “Oh dear - you treat me like a child!” It is so difficult because you must treat her like a grown-up person and at the same time explain to her what you mean. We have used time to explain to her that when she has taken tests…. then they can say if they can try to give her dementia medication. We have discussed the advantages of using Exelon plaster with her.”"

Many were sensitive about being given too much detailed information as this was taken to be treated in a childish way. In this quote there is no evidence of the person being informed of the effects or side-effects of the medication. At times family carers and professional caregivers selected information to reinforce own opinions.

### Non-involvement

A main reason for non-involvement was that because of progressing dementia persons were no longer capable of being involved in active decision making. Another major reason was that people interacting with them did not give them the opportunity to be involved or that there were few or no choices available.

This is illustrated in the following case:

Every year Mr F and his wife travelled to Southern Europe where they especially enjoyed dancing in the evenings. He had recently fallen, resulting in a hip fracture and his wife was very concerned that their chosen life-style could not be continued. She said:

"“I give him directions and he does as he is told… I force him to train … and remind him to eat and drink because he forgets. I told him that if he didn’t get up and walk, he would be left sitting there in a wheel chair. Those are his choices!”"

The wife appears to have been the dominating partner in their relationship, and the situation was aggravated by his mental decline and his physical disability. She positioned him as incompetent and patronized him, adding to his distress. The underlying problem was that she had not accepted the situation and by threatening him to train, she might have hoped that his condition would return to “normal” and that their life continue as before.

"In another case Mr A was taken to the physician by his wife for diagnosis. He did not understand the information he was given. He said there was something wrong with his wife and that she was the one who needed her head examined. According to his Neuropsychiatric Inventory score, he was aggressive and restless at times."

"Later, in the nursing home, he was restless and appeared to be very upset, constantly asking for his wife and wanting to leave the premises. He expressed clearly that he was being kept there against his will. He said his wife lied about why he had to be there and she threatened to stop visiting him if he did not stop nagging about coming home. The caregivers did not take him seriously but teased him, asking him if he really wanted to leave them. Since he may have feared seeming impolite, he was not likely to say openly that he did not want to be there."

This case illustrates the need to understand each situation in its context. Mr A did not know the reasons for major decisions taken on his behalf. This added to his confusion and unrest, especially being admitted to the special care unit with locked doors.

But could there be some grounds for his suspicion? Without consulting him, his wife sold their house, moved to another municipality, bought a new flat, stopped giving him his medicine, took their dog to the vet to be put to sleep and arranged for his transfer to a nursing home because of her own health problems. She said that earlier on in their married life he had made all the decisions and now it was her turn to decide and pursue her own interests. Mr A’s non-involvement could be explained by how his wife made decisions that left him disempowered, frustrated and resigned.

The professional caregivers could perhaps have tried to explain why his wife’s illness made it necessary for him to be there in the hope of him understanding, although partially, that there were no other alternatives, and helping him accept and adjust to his present life. This case is complicated by the autonomy of two persons feeling threatened. In this case the staff appeared to allow the wife a superior stance over Mr A in decision making, thus eroding his rights. In addition, this case illustrated there are boundaries and limits for decision making as it was no longer a realistic alternative for Mr A to return home.

There were at times a glaring gap between ideals and realities. In an interview with a primary caregiver she said that in the nursing home residents could decide when they wanted to get up or go to bed, what to eat or if they wanted to participate in special activities. Observations during breakfast revealed that it was not quite so. Residents were seated at tables with prepared food on their plates. There was no opportunity to choose spread and there was no offer of more food or coffee. Mrs D was underweight but had a good appetite. She said she had not asked for more because the staff was so busy and did not pay her any attention. In this situation, disinterested caregivers and understaffing prevented caregivers facilitating decision making.

## Discussion

The aim of this study was to understand how persons with dementia participated in decision making related to health and daily care and how family carers and professional caregivers influenced decision making processes.

### Assessment of competence

Evidence of assessing the person’s mental capacity was scarce even though all had taken a Mini-Mental-Status-Examination [50]; not a completely reliable test and therefore recommended to be used in conjunction with other tests [66] Only in one case (Mrs J) was there an assessment based on guidelines suggested by Grisso and Appelbaum [20], Lai and Karlawish [67], Mitty [68] and Walaszek [69]. When assessing mental competence, Engedal and Kirkevold [70] claim that health workers in Norway rely more on experience than on using standardized tests. This is a challenge in view of the fact that decisions with high-risk consequences require greater competence and more stringent assessment [18].

There is a growing recognition that competence is not a question of all or nothing but is spread across a wide continuum [71]. Competence can vary according to circumstances as well as how the person is feeling that day and how the person is understood and treated [1,7]. Appraisal of competence on a day-to-day basis taking the particular decision and relationships into consideration is vital. Maybe it is not so much a question of being either competent or incompetent but rather assessing the degree of competence along a continuum. This allows for persons being able to make some decisions but not others.

The focus on determining individual competence for decision making needs to be shifted to how persons with dementia can be empowered to participate in decision making processes irrespective of cognitive functioning [22]. This entails knowing the person and identifying retained abilities, promoting understanding, appreciation, reasoning and expressing choice in addition to being aware of what impact values and relationships have on decision making.

### Variability in how persons with dementia participate in decision making

A major finding was that persons with moderate dementia were involved in decisions, supporting several studies [1,29]. In earlier times, the voices of persons with dementia were not heard because unquestioned assumptions of incompetence led others to make decisions on their behalf [72]. This resulted in a culture of “therapeutic nihilism” and custodial care [73]. In contrast, persons in this study demonstrated how they participated in decision making in various ways, giving new perspectives on how to improve dementia care.

Findings from this study expand on existing work by describing new and different levels of involvement not identified by Thompson [45] such as “Pseudo-autonomous decision making” and “Delegating decision making” when persons with dementia were involved in decision making.

Pseudo-autonomous decisions were implicit rather than explicit and often rooted in family members or health care workers falsely assuming that they knew the wishes of persons with dementia and acted accordingly. As cognitive abilities decline, understanding relationships and clarifying values seem to take on added importance. In Thompson’s study participants probably relied more on their cognitive abilities. In addition Thompson focused on the individual patient’s participation and not so much on relational aspects in decision making.

By delegating responsibility to others, persons with dementia demonstrated their dependence on others. The literature offers several reasons for delegating authority: awareness of cognitive decline and fear of making wrong decisions [74]; desire to live up to expectations of being a “good” patient by deferring all decision making to persons with authority [75]; older people are more likely to acquiesce and submit to authority [76]; and delegating as a compensatory response to optimize function in other domains [77]. Similar reasons were identified in these data but there was no evidence for concluding that delegating authority was a compensatory response.

In this study the category “Shared decision making” combined Thompson’s three categories of “Information seeking/information receptive”; “Information dialogue”; and “Shared decision making” because it was difficult to separate and identify these specific ways of participating in dementia care.

More autonomous decisions were made about daily activities than decisions about medical treatment and moving. This was most probably because decisions about daily care are fairly predictable and without serious consequences. In major decisions, sharing responsibility for making decisions was the main pattern. The ideal of autonomous decision making can be unrealistic when it comes to certain complex decisions and obscures the basic interdependence and necessity of shared decision making. However, autonomous decision making cannot be ruled out all together as in the case of Miss G who wished to remain living in her own home.

### The importance of relationships and context

The study underlined the importance of understanding decision making in a relational context. The right to participate in decision making in health care raises issues about sharing power in patient-caregiver relationships [78,79]. There is a real possibility of the family carers or health professionals controlling the conversational agendas and dominating decision making. Professionals have knowledge that puts them at an advantage when defining what is medically best for the person with dementia, causing a power imbalance in the relationship and causing professionals to feel justified in making decisions. Information can be manipulated by withholding data or using information to reinforce decisions already taken by others. Exercising such power was seen in cases where caregivers tried to convince persons with dementia to see things their way.

Another main finding was that family bonds characterized by affection and norms of reciprocity or tensions and power-struggles influenced decision making. With dementia, roles and power-dynamics within a relationship changed. At times families can confuse what is in the patient’s best interests with their own interests [28].

Health professionals can more actively seek the opinions of the family rather than those of the person with dementia [15] or empathize with family members and form alliances against the person with dementia or exclude them from making decisions [13,14]. Another possibility is that the person’s wishes are overridden by the wishes of the family in spite of support from professional caregivers.

In the face of such conflicts McCormack [42] suggests applying a framework of negotiation based on the person’s values. This implies that clarifying values and integrating triadic perspectives allows for different opinions to be discussed until consensus is reached. The right to participate in decisions needs to be adapted to the realism of interdependency in dementia care. The person with dementia is not always the best judge of their interests and family members might have legitimate issues needing resolution. The responsibility of health professionals is to balance needs and facilitate decision making through optimal participation of the person with dementia.

With the progress of dementia, persons with dementia were satisfied with letting others take over responsibility for them. Not being given opportunities to participate could have adverse effects, e.g. Mr A in the nursing home. His problems were not only due to his dementia but also to difficult interactions with his wife and other caregivers. Individual measures such as medicating Mr A would thus not have been an adequate solution to his situation. His disability was not only rooted in his brain but also in the social world where he was constructed as dysfunctional.

Decision making competence of persons with dementia was affected by the culture of care, the staff’s behaviour and budgetary and structural constraints registered in a study by Helgesen [80]. This was also the case in this study. Even though the legal right to participate in decision making was part of the explicit ideology of the health services, these ideals were difficult to implement in practice. Health care professionals reported of institutional objectives being prioritized over the needs of persons needing services. Inadequate staffing, high turnover and no continuity of care resulted in depersonalized and task-oriented care giving. These conditions made it difficult to get to know the person with dementia and to work in partnership with family carers. This aligns with results in a study by Kirkevold and Engedal [81], where it was found that ward characteristics such as wards for persons with low function in mental capacity, size of ward and staffing ratio influence the quality of care.

#### Strengths and limitations of the study

The strength of this multi-case study is that it offered in-depth descriptions and interpretations increasing our understanding of nuances in decision making in dementia care. Persons with moderate dementia had a variety of family carers with whom some had close and others more strained relationships. They also lived in different settings, varying from being a patient in a special unit in a nursing home to living independently in their own home. The small sample is a limitation so there may be patterns of participation that have not yet been discovered. The findings cannot be generalized to apply to all persons with dementia but this study has identified that these patterns of involvement in decision making do exist.

## Conclusions

Persons with moderate dementia demonstrated variability in how they participated in decision making. Optimal involvement was facilitated by positioning them as capable of influencing decisions, assessing decision-specific competence, clarifying values and understanding the significance of relationships and context.

## Competing interests

The authors declare that they have no competing interests.

## Authors’ contributions

KLS had the main responsibility for conception and design, acquisition of data, analysis and interpretation of data in addition to drafting the manuscript. MK contributed to the conception and design of the study, analysis and interpretation of data and revising the article critically. KE was involved in the analysis of data and in revising the article critically.

## Pre-publication history

The pre-publication history for this paper can be accessed here:

http://www.biomedcentral.com/1472-6963/12/241/prepub
